# Direct fabrication of 3D graphene on nanoporous anodic alumina by plasma-enhanced chemical vapor deposition

**DOI:** 10.1038/srep19822

**Published:** 2016-01-25

**Authors:** Hualin Zhan, David J. Garrett, Nicholas V. Apollo, Kumaravelu Ganesan, Desmond Lau, Steven Prawer, Jiri Cervenka

**Affiliations:** 1 School of Physics, The University of Melbourne, Parkville, VIC 3010, Australia; 2 The Bionics Institute, 384-388 Albert Street, East Melbourne, Victoria 3002, Australia; 3 School of Applied Sciences, RMIT University, Melbourne, VIC 3001, Australia; 4 Institute of Physics ASCR, v.v.i., Cukrovarnická 10, 16253 Praha 6, Czech Republic

## Abstract

High surface area electrode materials are of interest for a wide range of potential applications such as super-capacitors and electrochemical cells. This paper describes a fabrication method of three-dimensional (3D) graphene conformally coated on nanoporous insulating substrate with uniform nanopore size. 3D graphene films were formed by controlled graphitization of diamond-like amorphous carbon precursor films, deposited by plasma-enhanced chemical vapour deposition (PECVD). Plasma-assisted graphitization was found to produce better quality graphene than a simple thermal graphitization process. The resulting 3D graphene/amorphous carbon/alumina structure has a very high surface area, good electrical conductivity and exhibits excellent chemically stability, providing a good material platform for electrochemical applications. Consequently very large electrochemical capacitance values, as high as 2.1 mF for a sample of 10 mm^3^, were achieved. The electrochemical capacitance of the material exhibits a dependence on bias voltage, a phenomenon observed by other groups when studying graphene quantum capacitance. The plasma-assisted graphitization, which dominates the graphitization process, is analyzed and discussed in detail.

Ever since the first experimental proof of graphene’s existence^1^, graphene has attracted immense interests, primarily due to its unique atomic^2^ and electronic^3^,^4^ two-dimensional (2D) structure. The electronic properties of graphene, in particular, are intensively researched for applications as components in next generation electronics^5^, high-performance electrochemical energy storage^6^,^7^,^8^,^9^,^10^,^11^ and sensors^12^. In super-capacitors^6^,^7^,^8^,^9^,^10^,^11^ and electrochemical biosensors^12^, graphene has a large advantage compared to other materials owing to its atomic thickness and ability to generate large specific surface area macrostructures^8^,^12^,^13^. However, fabrication of three dimensional (3D) graphene grown over well-defined nanopores and atomic thickness is not straightforward.

Previous fabrication processes related to producing 3D graphene films on porous substrate have usually involved multiple fabrication steps and required the use of graphene oxide^11^,^12^,^13^,^14^,^15^,^16^. Graphene oxide, however, needs to be chemically activated to achieve well-conductive graphene films^15^ and the 3D graphene made of it suffers from undefined porous structure with broad distribution of pore sizes^13^,^14^,^15^. Other fabrication approaches using chemical vapour deposition (CVD) have required the use of metal catalysts^13^,^14^, which needed to be etched away to allow its use in electrochemical applications. For this reason it would be more suitable to grow graphene on porous dielectric substrates. Porous dielectric materials have been intensively researched in the past decades and offer well-defined ordered pores with a great range of sizes, shapes and porosities^17^,^18^,^19^. Although growth of 3D amorphous carbon on ordered porous dielectrics has been previously demonstrated with^20^,^21^,^22^ and without^23^ catalyst, catalyst-free direct 3D graphene growth on ordered porous dielectrics has not been previously reported to our knowledge.

This paper presents a technique for one-step graphene fabrication on nanoporous anodic aluminium oxide (AAO) using plasma-enhanced chemical vapor deposition (PECVD). We demonstrate direct catalyst-free graphene growth on AAO and investigate the formation of graphene in the nanopores. High effective surface area graphene grown over well-defined nanopores with large capacitance (as high as 2.1 mF for a sample of 10 mm^3^ with the effective surface area of 883 cm^2^) is achieved. The material properties of 3D graphene are analyzed by Raman spectroscopy, Scanning Electron Microscopy (SEM), Transmission Electron Microscopy (TEM) and Electron Energy Loss Spectroscopy (EELS). The electrical and electrochemical properties are investigated by conductance measurements, Cyclic Voltammetry (CV), and Electrochemical Impedance Spectroscopy (EIS). The chemical stability of the material is studied by chemical etching using hydrofluoric acid (HF). Based on the results of the material characterization and analysis of the plasma-material interaction, it is concluded that the growth mechanism is a combination of deposition and graphitization of an ultrathin amorphous carbon layer on AAO in PECVD.

## Results

**Figure 1:**
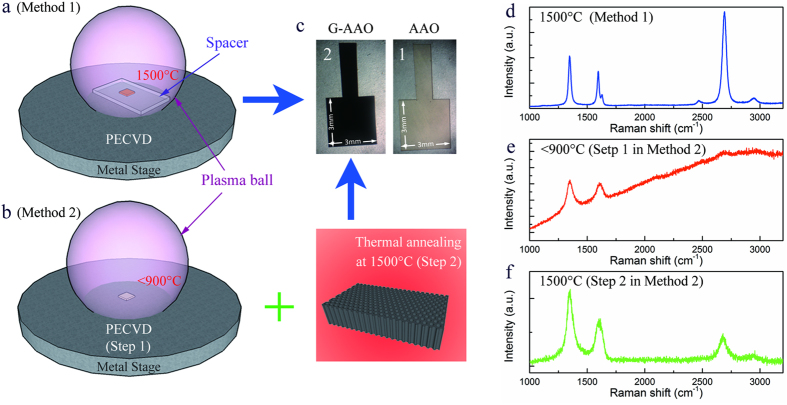
Fabrication processes and Raman spectra of 3D graphene and a-C on AAO. (**a**) Schematics of the one-step graphene fabrication method by PECVD process with the spacer in between the sample and the stage. (**b**) Schematics of the two-step method where the 1^st^ step is the PECVD process without the spacer and the 2^nd^ step is thermal annealing of the a-C-AAO sample (produced by step 1) in vacuum at 1500 °C using an electron beam. (**c**) An AAO sample before (1) and after (2) the fabrication process. (**d**–**f**) Raman spectra of graphene coated AAO (G-AAO by method 1), diamond-like carbon coated AAO (a-C-AAO by the 1^st^ step in method 2) and thermal annealed a-C-AAO (by the 2^nd^ step in method 2), respectively.

Figure 1 presents two fabrication methods for 3D graphene on insulating nanoporous AAO substrates using one-step and two-step method in PECVD. In method 1, namely the one-step graphene fabrication method (Fig. 1a), a quartz spacer (0.35 mm thick) was used to electrically and thermally isolate the AAO from the molybdenum growth stage during the growth. This method allowed one-step graphene production on AAO with an interlaying thin layer of amorphous carbon (a-C) and reaching substantially higher substrate temperatures (≈1500 °C) in the PECVD process than without the spacer. In the two-step fabrication method (method 2 as shown in Fig. 1b), an ultrathin a-C layer was first grown on AAO without the spacer using the same reactor conditions as in method 1. The temperature of the sample was below 900 °C during the a-C growth process. To obtain graphene on a-C-AAO samples, the sample was further annealed at the temperature of 1500 °C in vacuum.

Figure 1c shows an optical image of AAO sample before (1) and after (2) the graphene PECVD process. The samples produced by method 1 and 2 both became black after the process because of the light adsorption in graphene on the pore walls in the sample (5 × 10^9^ cm^−2^ pore density). Unless specified otherwise, the following characterizations are made based on the sample fabricated by method 1.

### Raman spectroscopy

Figure 1d shows a Raman spectrum of a graphene-coated AAO (G-AAO) sample using method 1. The main features in the Raman spectrum of G-AAO are the typical graphene related D (1348 cm^−1^), G (1595 cm^−1^) and 2D (2691 cm^−1^) peaks. The rise of D and D’ (1629 cm^−1^) peaks suggest the existence of defects in the sample^24^. The intensity ratio between D and G peaks (I(D)/I(G)) is 1.5, which indicates disorder and the presence of sp^3^ type defects^24^,^25^,^26^,^27^, most probably originating from grain boundaries in the graphene layers. Using the intensity ratio between D and G peaks, which is a common method used to analyze disorder in graphitic samples^25^,^26^,^28^, we estimate the average graphene grain size as 
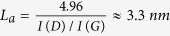
 for the 532 nm laser excitation. The full-width-at-half-maximum of the 2D peaks (FWHM(2D)) of the sample is 41.9 cm^−1^, which is slightly larger than that of a commercially purchased CVD monolayer graphene on SiO_2_ (32.3 cm^−1^) consisting of a single 2D component but lower than of bilayer graphene (50 cm^−1^), which contains four 2D peak components^29^. This suggests that the sample is composed predominantly of monolayer graphene and the slight broadening of the 2D graphene peak could be explained by the defective nature of 3D graphene and a large number of grains in the focal spot size. The much greater intensity of 2D peak with respect to the G peak is also consistent with a dominant contribution from single layer graphene or rotated multilayer (non Bernal stacked) graphene flakes^16^,^27^,^30^.

Figure 1e depicts the Raman spectrum of a sample grown without a spacer, namely by method 2, and is typical of diamond-like amorphous carbon-coated AAO (a-C-AAO)^16^,^31^,^32^. This sample was additionally annealed in vacuum at 1500 °C as shown in Fig. 1b. The thermal annealing of a-C-AAO has led to improved crystallinity (indicated by sharper G and 2D peaks in the Raman spectrum in Fig. 1f) and an increased portion of sp^2^ carbon hybridization in the a-C-AAO samples^27^. FWHM(2D) of thermally annealed a-C-AAO is 80 cm^−1^ and the I(D)/I(G) is 1.8. The Raman spectrum indicates a defected few-layered graphene was obtained, confirming the temperature driven graphitization process^33^.

### SEM

**Figure 2:**
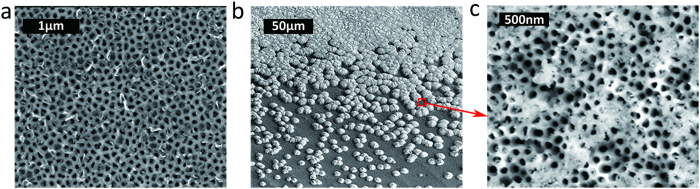
SEM pictures of G-AAO grown by method 1. (**a**) G-AAO with good porous structure. (**b**) Carbon overgrowth on AAO. (**c**) High magnification images taken from specific areas on (**b**).

Figure 2a shows SEM image of a surface of G-AAO grown by method 1, demonstrating that the porous structure of the alumina sample remains after the high temperature PECVD process. The SEM image of AAO before PECVD can be found in figureS1d in the supplementary file. Figure 2b and c shows that overgrowth (an appearance of bumps) can happen during the process at higher microwave power (3 kW) on the front side of the sample directly contacting the plasma. Interestingly, the Raman spectra of samples with overgrowth (front side) and no overgrowth (back side) look the same. The overgrowth layer also maintains porous structure, even though some pores are blocked. The overgrowth process has previously been observed during amorphous carbon growth on AAO by other groups^21^,^22^,^23^,^31^. The Raman spectrum of G-AAO with overgrowth (which is identical to Fig. 1d) indicates much higher sp^2^ content in our study than in previous reports^23^,^31^,^34^. More detailed microscopic analysis of the overgrowth can be found in the supplementary file.

### TEM and EELS

**Figure 3:**
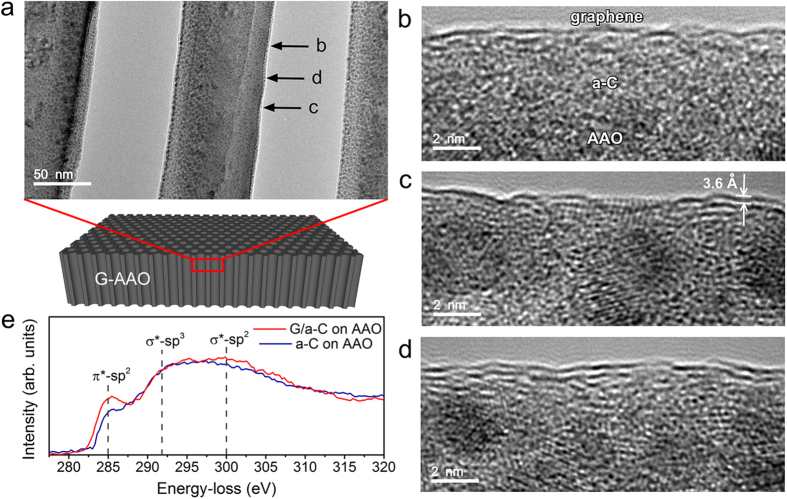
TEM and EELS analysis of G-AAO produced by method 1. (**a**) Cross-sectional TEM picture of the porous structure inside G-AAO, while the schematics below illustratively indicates the location analysed. (**b-d**) High magnification images taken from the corresponding regions in (a), which indicate monolayer graphene, a combination of monolayer and few-layered graphene (where the interlayer distance is 0.36 nm), and FLG, respectively. (**e**) A comparison of EELS results between G-AAO (red) and a-C-AAO (blue).

**Figure 4:**
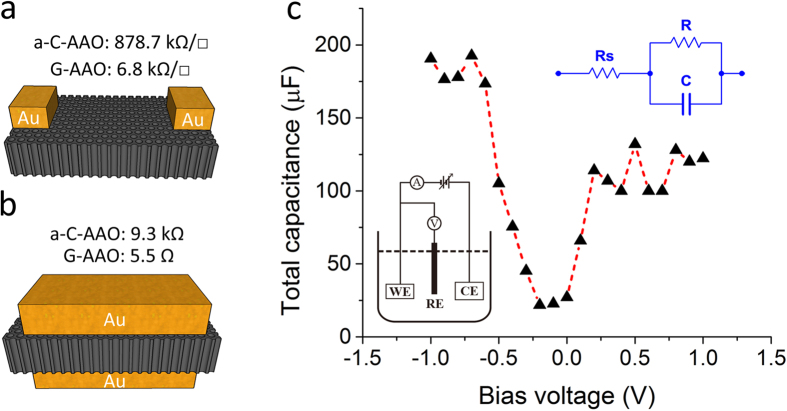
Electrical and electrochemical measurements. (**a,b**) Schematic illustrations for resistance measurement horizontally along and vertically across the sample, respectively, where the black porous membranes are the samples (a-C-AAO or G-AAO) and the golden blocks are the contacts. The values indicate the sheet resistance and resistance in (**a,b**), respectively. (**c**) The total capacitance variation of G-AAO with the bias voltage changing in ferrocyanide solution. The inset at the lower left corner depicts the three-electrode electrochemical cell, where WE, RE and CE represent working electrode, reference electrode and counter electrode. The inset at the upper right corner is the equivalent circuit (the Randles circuit) between the working electrode and the counter electrode used for evaluation of capacitance.

To analyze the atomic structure and a number of graphene layers in the samples, TEM studies of the G-AAO membranes have been performed. Figure 3a shows a cross-sectional TEM image of the nanoporous structure of G-AAO. The sample consists of graphene top layer, a-C interlayer and nano-crystalline alumina core, as shown in the high magnification images in Fig. 3b. The microscopic analysis of the structure of a-C on AAO has been reported previously in ref. 35. The number of graphene layers has been found to be inhomogeneous across the surface, showing co-existence of monolayer and few layer graphene (FLG) in the sample. This is demonstrated in Fig. 3b–d by high magnification images of the corresponding regions marked by arrows in Fig. 3a. Monolayer graphene is observed in Fig. 3b, while there is co-existence of monolayer and multilayer graphene in Fig. 3c and 3–4 layer graphene in Fig. 3d. Some graphene nano-sheets are bent, most probably due to the interatomic potential at the edge of the overlapping layers^36^. The interlayer distance between two layers is approximately 0.36 nm. This is the typical graphene interlayer distance in FLG^33^,^37^. The size of graphene flakes is of the order of few nanometers, which is similar to the estimated graphene grain size from the Raman spectroscopy study using I(D)/I(G).

Figure 3e shows carbon K-edge EELS spectra for G-AAO (red) and a-C-AAO (blue) taken at the surface of the nanopores using a focused electron beam (≈0.5 nm). The peak evident at the photon energy of 285 eV of G-AAO in Fig. 3e represents the π* resonance in carbon K-edge, which corresponds to a transition of electrons to unoccupied carbon π* states. Gaussian fitting to the EELS spectra indicates the sp^2^ fractions are 77.2% and 50.9% in G-AAO and in a-C-AAO, respectively, where glassy carbon (100% sp^2^) was used as the reference^38^,^39^. This increase in G-AAO provides a direct evidence of a-C graphitization. A strong σ*-sp^3^ peak in G-AAO supports the presence of a remaining amorphous a-C carbon layer underneath graphene layers

### HF corrosion test

To confirm the full coverage of the graphene layer over AAO, G-AAO was inserted in HF acid (concentration 40%, pH 3.5) for 22 hours to study the chemical stability of the material^40^. The sample remained the same after the HF corrosion test, suggesting the G-AAO sample is fully covered by the protective graphene layer, since exposed alumina would be etched away by HF.

### Electrical resistance

To test the continuity and electrical conductivity of the formed graphene layers on AAO we performed a two point probe electrical measurements. The sheet resistance measured horizontally along the surface, as schematically seen in Fig. 4a, showed significant decrease from a-C-AAO (878.7 kΩ/□) to G-AAO (6.8 kΩ/□), confirming the conductive nature of the 3D graphene. The electrical resistance measured vertically across G-AAO (5.5 Ω) was also much smaller than that of a-C-AAO (9.3 kΩ), as illustratively shown in Fig. 4b, confirming the nanopores are all coated with graphene. Note that all the sample edges of G-AAO and a-C-AAO have been cut off in the vertical measurements in order to obtain the resistances contributed from the pores only, and the smaller resistance values of vertical contacts compared to horizontal are due to the millions of conductive channels/pores connected in parallel.

### Electrochemical capacitance

Electrochemical capacitance (Fig. 4c) of a G-AAO sample (3 mm × 3 mm × 0.1 mm as shown in Fig. 1c) was measured by electrochemical impedance spectroscopy (EIS) analysis using a three-electrode electrochemical cell^41^ (1 mM ferrocyanide 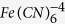 with 0.1 M KCl as the supporting electrolyte). The bias voltage was applied between the working electrode and the reference electrode. The measured total capacitance includes contributions from the parasitic capacitance, the graphene quantum capacitance and the electrical double layer (EDL) capacitance. Component values were estimated by fitting impedance data to the simplified Randles circuit^42^ (inset Fig. 4c), where C is the total capacitance, R_s_ is the solution resistance and R the resistance to charge transfer to 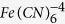. As shown in Fig. 4c, the total capacitance is found to be dependent on the bias voltage and it reaches a minimum at the voltage around −0.1V. This dependence has also been observed by other groups due to graphene quantum capacitance^43^,^44^ and electric double layer capacitance at potential of zero charge^41^.

Table 1 compares the specific capacitance and the overall capacitance of G-AAO (1 cm × 1 cm × 0.1 mm) with the same-sized gold electrode^45^,^46^, which is a commonly used bio-capacitor. The G-AAO and gold electrodes were tested in 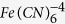 solution and biased at −1 V. It can be seen from the table that although the specific capacitance of G-AAO is smaller than that of gold, the overall capacitance is much larger for the G-AAO electrode due to its much larger surface area. This result suggests that due to this large capacitance G-AAO material may be used as a super-capacitor material in applications such as energy storage or neuronal cell stimulation.

## Discussion of the growth mechanism

The two presented fabrication methods of 3D graphene described in Fig. 1: (a) One-step plasma-driven graphene growth on AAO in PECVD and (b) Two-step method using thermal post annealing of a-c-AAO, suggest that the high temperature of the order of 1500 °C is crucial for graphene formation on AAO. Since it has been proved that a conformal a-C layer is formed on AAO before reaching this temperature^21^,^22^,^23^,^34^,^35^ (step 1 in method 2) and it can be thermally graphitized into graphene at 1500 °C (step 2 in method 2), the growth mechanism for graphene formation on AAO by method 1 can also be considered as a process graphitizing an ultrathin a-C layer. Thermal graphitization of amorphous carbon is a process that has been well studied^33^,^47^. Converting amorphous carbon to graphite starts at 1200 °C and is completed at 1600 °C, leading to complete transformation of the whole material to graphite. Raman spectroscopy has, however, shown a significant difference between G-AAO samples directly grown in PECVD and produced by post thermal annealing, demonstrating that the direct PECVD growth can lead to better quality graphene. This shows that hydrogen/methane plasma in direct PECVD growth plays a very important role^48^ and can improve the quality of graphitized graphene layers. γ-Al_2_O_3_ and α-Al_2_O_3_ formed at high temperature^49^ in AAO are also believed helpful for graphene nucleation and growth^50^,^51^. It is suspected that γ-Al_2_O_3_ and α-Al_2_O_3_ were involved in the plasma-enhanced graphitization process to form graphene due to the ion bombardment on the surface by energetic particles, while in thermal annealing γ-Al_2_O_3_ was well separated by the a-C layer from the surface graphene layer. The detailed a-C/γ-Al_2_O_3_/AAO structure can be found in ref. 35.

It has also been observed in the experiments that the growth of graphene or a-C in PECVD can be effectively controlled by a proper choice of a spacer under the sample, while using the same plasma growth conditions. The use of dielectric spacer allowed direct production of graphene on AAO and reaching 600 °C higher sample temperature of AAO on a dielectric spacer compared to a metal stage. The ability to control the sample temperature by the spacer in the PECVD process was found also extremely important for graphene production reproducibility. In some occasions, it was possible to obtain G-AAO without separating the AAO from the stage but the repeatability was low. Only one experiment out of ten could successfully produce G-AAO without the spacer.

### Thermal isolation

Intuitively, the dielectric spacer plays two roles that both lead to an increase of the temperature of the sample in the plasma. Firstly, the spacer introduces thermal isolation between the sample and the stage and therefore reduces the thermal dissipation of the sample. Secondly, the spacer induces perturbation to the plasma near the sample surface, which can lead to complicated plasma-material interactions and a sample temperature change, because plasma is the only source ‘heating up’ the sample (plasma-enhanced graphitization). The increase in sample temperature has also a significant impact on the surface chemistry, which can strongly affect the carbon layer formation.

For the quartz spacer of 0.35 mm thick, our calculation using Fourier’s law^52^ estimated that the thermal isolation effect contributes to around 45% (i.e. 270 °C) of the observed sample temperature increase by the spacer. This suggests that the role of plasma-induced heating is significant and it contributes to more than a half of the total sample heating in PECVD. It should be noted that the thermal isolation effect itself, however, can be very large for thicker spacers, for example the sample temperature can increase by 825 °C for a 1 mm thick spacer.

### Plasma-enhanced graphitization

Analysing all the chemical processes in plasma including plasma-material interaction, and hence simultaneously tracking every charged particles collision event, requires massive Monte-Carlo simulations of plasma^53^,^54^. This is normally conducted for tokamak fusion plasma^55^ but it is not realistic for our experiment. However, since the chemical processes and energy changes which contribute to thin film formation usually happen on substrate surface^56^, analysing the energy loss of the charged chemical species (ions) on substrate surface can be used as a good indicator of the temperature change. The kinetic energy of the ions (*E*_*k*_) on substrate surface is mainly determined by the electric potential in plasma sheath and provides the energy for ions to bombard the substrate surface. Ions move directionally within the plasma sheath driven by the potential drop^53^,^57^. For microwave plasma, *E*_*k*_ can be roughly estimated as *E*_*k*_  ≈ 5.2*T*_*e*_ ≈ 26 *eV*^56^, where *T*_*e*_ ≈ 5 *eV* is the electron temperature^58^. This gives *E*_*k*_ a value greater than the energy released in chemical processes, for example, the hydrogen binding energy (around 4.5 eV)^59^. Therefore the following analysis is focused on qualitatively investigating the factors which change the kinetic energy of ions, i.e. the potential distribution.

The ion bombardment is believed important in PECVD^60^. However, unlike direct-current gas charging, generating plasma by microwave does not require any bias between the plasma and the stage, and the metal stage in microwave PECVD chamber is grounded, so the potential drop within the sheath depends only on the plasma potential. Since the plasma potential is not necessarily zero and it varies with the external factors (such as the reflected microwave power and so on), the resulting unstable sheath potential on metal stage leads to different ion bombardment in every experiment. This could explain low repeatability of the same production conditions without spacer.

**Figure 5:**
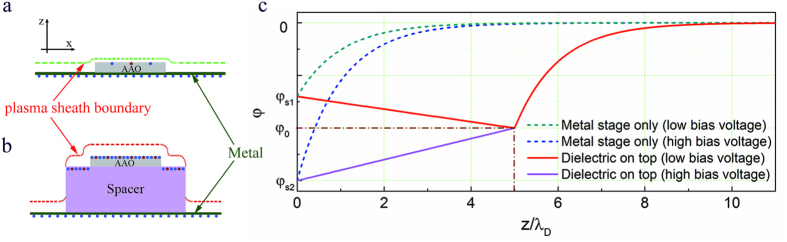
Plasma sheath potential distribution of AAO with and without spacer. Schematic illustrations of the plasma sheath formation on AAO directly sitting on top of (**a**) a metal stage and (**b**) a dielectric spacer on the metal stage, respectively. (**c**) Potential distributions as a function of distance away from the metal stage directly (dashed lines) and from the dielectric on the stage (solid lines) at different bias voltage.

By solving Poisson-Boltzmann equations^53^,^57^ with different boundary conditions, we obtained the potential distribution near the surface of AAO, i.e. inside plasma sheath, with and without the spacer as shown in Fig. 5 and discussed in detail in the supplementary material. The difference between whether or not the dielectric spacer is sitting on the metal stage is that the dielectric has the ability to re-establish its own ambipolar diffusion regardless of the potential of the stage due to surface charge accumulation, and therefore stabilizes the potential drop in the sheath. For the case represented by Fig. 5a, the unstable potential distributions at different bias voltage (where 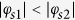) are indicated by the green and blue dashed curves in Fig. 5c. For the case represented by Fig. 5b, when a dielectric (with a thickness of 5*λ*_*D*_, where *λ*_*D*_ is Debye length) is placed on the top of the metal stage, the stable ambipolar field is created on the dielectric surface for both low and high bias voltages, as demonstrated by the red and purple solid curves in Fig. 5c, where *φ*_0_ is the potential determined by the ambipolar field. Therefore placing the dielectric on top of the metal provides a more stable plasma sheath than on the metal. The stable sheath offers continuous ion bombardment on AAO hence increases the temperature.

Although AAO itself is a dielectric, a rough estimation gives *λ*_*D*_ ≈ 53 *μm* if *T*_*e*_ = 5 *eV* and the electron density is 10^11^ cm^−3^ in microwave plasma source^58^. Hence the plasma sheath (usually a few Debye lengths^53^,^56^) is comparable or even greater than the membrane thickness, which makes the stabilization effect of AAO negligible (without including the porous nature of AAO). Therefore a relatively thick dielectric spacer was inserted beneath the AAO to create its own sheath without being affected by the grounded metal stage.

Another possible effect on temperature increase is surface charging, namely the accumulation of charges on the dielectric surface in plasma. Although the potentials on dielectric and on metal at far distance are found to be similar due to the same amount of net charges (Fig. S2 in Supplementary data), the density of the total charged particle number is much larger on dielectric (by a factor of 3, 5, or even more). When there are more charged particles accumulating on the dielectric surface due to the diffusion and the potential drop in the plasma sheath, the complex electric field near the surface keeps increasing until the breakdown field is reached, and then heat is released. This process can be viewed similar to graphitization induced by current annealing^47^, and will contribute to annealing/heating up of the sample. It is speculated that this process is more effective in the stable electrical environment created by the dielectric spacer, since the unstable potential sheath on metal will affect the charge accumulation on AAO.

## Conclusions

The possibility of catalyst-free graphene growth on a porous dielectric AAO material was explored. Two different fabrication methods of graphene on AAO were developed based on plasma-enhanced and thermal graphitization of ultrathin a-C precursor layers in PECVD. Microscopic studies confirmed that defected FLG is conformally obtained on the whole internal and external surface of nanoporous AAO. The samples retained the well-defined nanoporous structure of the AAO and become well conductive after the graphene deposition process. Overgrowth of G-AAO and occlusion of pores was an intermittent problem, which can be avoided by using lower plasma power. Due to the large surface area of the porous structure, the electrochemical capacitance as large as 2.1 mF for the sample of 10 mm^3^ with effective surface area of 883 cm^2^ was achieved. This implies the potential for using G-AAO as super-capacitors for energy or/and biological applications. The capacitance dependence on the bias voltage of G-AAO was also observed.

EELS and Raman strongly suggest that plasma-enhanced graphitization of a-C is the growth mechanism of G-AAO in PECVD. To improve the reliability of the G-AAO film production and achieve higher substrate temperature in PECVD for production of graphene, the sample plasma annealing process was investigated by studying the surface charging effect and the potential distribution on the plasma-material interface. It was found that introducing a dielectric spacer in between the sample and the metal stage leads to 600 °C higher sample temperature and improved repeatability than on the metal stage due to several reasons, such as stabilization of the plasma sheath, surface charge accumulation for direct annealing, and reduction of the power dissipation.

## Methods

AAO samples with 55 nm pore diameter and 100 μm thickness (Synkera Technologies, Inc.) were placed within the growth chamber of a microwave PECVD system (iplas GmbH). Graphene (the one-step method) and a-C (the first step in the two-step method) were grown on AAO using the following reactor conditions: H_2_/CH_4_: 750/10 sccm; microwave power: 1.7 kW; pressure: 85 Torr. The temperature of the samples was monitored by an external pyrometer outside the chamber and a thermocouple in the molybdenum stage. Assistive stage heating prior to the PECVD process was used to prevent the quartz spacer from breaking due to the sudden temperature rise coming from plasma. Raman spectroscopic studies were conducted on a Renishaw inVia Raman Microscope with a laser wavelength of 532 nm and a laser spot size of 10 μm. SEM analyses were performed on FEI Nova Nanolab 200. TEM and EELS characterizations were conducted on a JEOL 2100F. The electron energy applied in EELS studies was 80 keV, and the collection angle was 16 mRad. 5 samples were tested in electrical and electrochemical measurements. Electrochemical impedance spectroscopy (EIS) data was acquired using an eDAQ z100 Electrochemical Impedance Analyzer connected to an eDAQ EA163 potentiostat. The frequency range in the EIS measurement was set from 1 kHz to 100 kHz, and the amplitude of the AC signal was kept as small as possible (30 mV) to minimise quantum capacitance effects. Capacitances values were estimated by fitting EIS data to a model circuit using ZMan v2.2 software (Zive Lab, WonATech).

## Additional Information

**How to cite this article**: Zhan, H. *et al*. Direct fabrication of 3D graphene on nanoporous anodic alumina by plasma-enhanced chemical vapor deposition. *Sci. Rep.* **6**, 19822; doi: 10.1038/srep19822 (2016).

## Supplementary Material

Supplementary Information

## Figures and Tables

**Table 1:** Comparison of the capacitance between G-AAO and gold bio-capacitors with the size of 1 cm × 1 cm × 0.1 mm.

Capacitors	Specific Capacitance	Effective surface area	Overall Capacitance
G-AAO	2.4 μF/cm^2^	882.98 cm^2^	2117 μF
Gold^45^,^46^	9 μF/cm^2^	1 cm^2^	9 μF
