# Supplementary material for: Direct fabrication of 3D graphene on nanoporous anodic alumina by plasma-enhanced chemical vapor deposition
Source: Sci Rep. 2016 Jan 25;6:19822. doi: 10.1038/srep19822 (PMC4726322; doi:10.1038/srep19822)
Supplement: Supplementary Information [file srep19822-s1.pdf]

Supplementary Information

Direct fabrication of 3D graphene on nanoporous anodic alumina by plasma-enhanced chemical vapor deposition

Hualin Zhan[1,], David J. Garrett[1,2], Nicholas V. Apollo[1], Kumaravelu Ganesan[1], Desmond Lau[3], Steven Prawer[1] and Jiri Cervenka[1,4,]**

[1] School of Physics, The University of Melbourne, Parkville, VIC 3010, Australia

[2] The Bionics Institute, 384-388 Albert Street, East Melbourne, Victoria 3002, Australia

[3] School of Applied Sciences, RMIT University, Melbourne, VIC 3001, Australia

[4] Institute of Physics ASCR, v.v.i., Cukrovarnická 10, 16253 Praha 6, Czech Republic

Supplementary SEM images for AAO and G-AAO

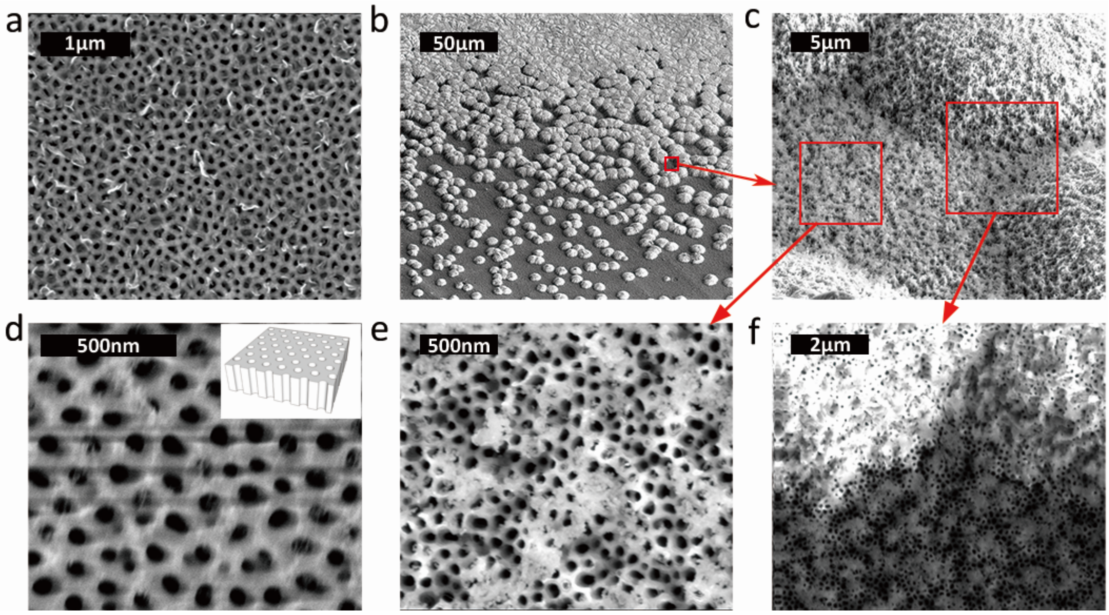

Figure S1. *SEM pictures of AAO and G-AAO.* **a**, G-AAO with good porous structure. **b**, Carbon overgrowth on AAO. **c**, High magnification images taken from specific areas on (b). **d**, AAO before graphene growth (inset: an illustrative schematic of AAO indicating the pores going through) **e,f**, High magnification images taken from specific regions on (c).

Figure S1d is the SEM image of AAO before graphene growth. The lines on the image were caused by the charging effect of AAO in SEM. Figure S1a shows the porous structure of G-AAO without overgrowth layer, while Figure S1b shows overgrowth on AAO. Figure S1c is a high magnification image taken from Figure S1b. Figure S1e and f are high magnification images taken from Figure S1c with and without the overgrowth bumps, respectively. Note that Figure S1b and c are taken when the sample was tilted at 52 °, while the rest are taken directly from the top of the sample. It can be seen clearly from Figure S1e that the porous

**Corresponding authors.*
Email addresses: h.zhan1@student.unimelb.edu.au, jiri.cervenka@gmail.com

nature of the sample remains, even though some pores are blocked by overgrowth. Figure S1c and f shows that the 'bumps' also retain the porous structure of the AAO.

Potential distribution for charged particles on metal and on dielectric

Let us consider electrons (blue spheres) to be distributed in a line evenly in free space as shown in Figure S2a. The electrical potential can be calculated as plotted in Figure S2c by solving Poisson equation. If we add ions (the red spheres in Figure S2b) in the same line but keep the net charge the same, it is seen from Figure S2d that the calculated potential is much more complex close to the charge line than in larger distance. In practice, Figure S2b resembles a negatively charged floating metal sheet because ions can move freely on the surface and cancel out part of the contribution for potential from electrons. While on a dielectric surface described by Figure S2b, all the charged particles are trapped and cannot simply counteract each other. It is worth noting and emphasizing that although the potentials are drastically different close to the surfaces in Figure S2a and b, the potential distributions are still similar at relatively far distance.

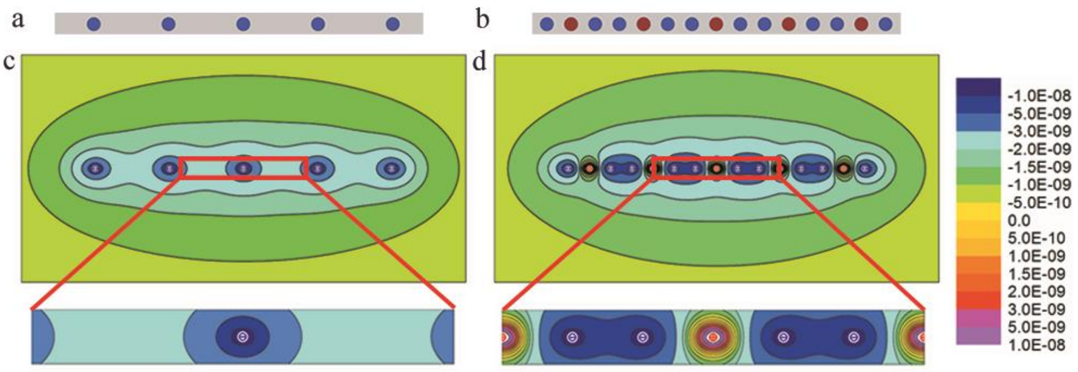

Figure S2. Calculated electrical potential distribution (shown by (c) and (d)) around lines composed of electrons (blue spheres) only (a) and of mixture of electrons and ions (red spheres) (b), respectively. Note the net charges are the same for (a) and (b). The scale bar represents the calculated electrical potential in arbitrary unit.

Plasma sheath formation on the dielectric sitting on the metal surface

Plasma sheath is a layer composed of an unbalanced number of differently charged particles formed on the material immersed in plasma due to the different mobility and diffusion rates between electrons and ions, causing a potential drop within the sheath. The sheath width is

usually of the order of several Debye lengths (λ_D). Depending on the bias voltage between the material and the plasma, the sheath can have different length and width.

1. The plasma sheaths on metal and on dielectric.

For an isolated material immersed in plasma, an ambipolar field will be established due to the ambipolar diffusion. At the steady state that ambipoblar diffusion reached, the electron flux is equal to the ion flux, i.e. $n_e \mu_e E_A - D_e \nabla n_e = n_i \mu_i E_A - D_i \nabla n_i$, where E_A is the ambipolar field, n_e and n_i are the electron and ion densities, μ_e and μ_i are the electron and ion mobility, D_e and D_i are the electron and ion diffusion coefficients, respectively. Since the electrical potential attenuates away from the material surface similarly for both metal and dielectric, the ambipolar field established will be similar. Therefore the charged particles are still described by Boltzmann distribution, thus the Debye length can still be found by solving Poisson-Boltzmann equations, which is $\lambda_D = \left(\frac{n_{e0} e^2}{\varepsilon_0 k_B T_e} + \frac{Z_i^2 n_{i0} e^2}{\varepsilon_0 k_B T_i}\right)^{-1/2}$, where k_B is the Boltzmann constant, n_{e0} and n_{i0} are the electron and ion densities in plasma, T_e and T_i are the electron and ion temperature, respectively. And the potential attenuation away from the metal surface is described by $\varphi = \varphi_0 exp\left(-\frac{z}{\lambda_D}\right)$, where φ_0 is the potential determined by the ambipolar field. It can be seen from the above flux equation that E_A only depends on the intrinsic properties of plasma and does not change with the external potential.

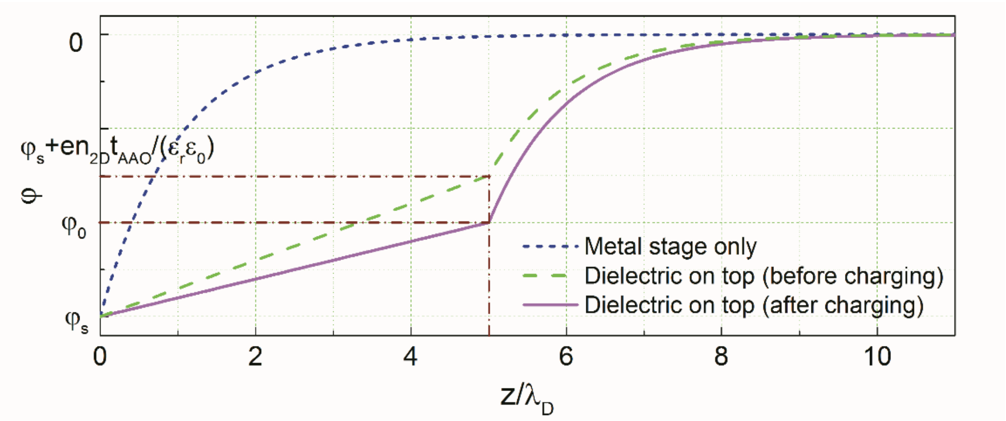

Figure S3. *The potential distribution in plasma.*

However metal in plasma is not necessary isolated and can be biased at different voltage, therefore $\varphi = \varphi_S exp\left(-\frac{z}{\lambda_D}\right)$, where φ_S is the biased voltage on the surface. The potential distribution is plotted as the blue dashed curve in Figure S3. The plasma sheath formed on the surface of the material immersed in plasma is often of several Debye lengths.

2. The plasma sheath on the dielectric sitting on the metal surface before the charges accumulate on the dielectric surface

To analyse this problem, it's convenient to start with the case where the sample was on an isolated metal stage (neither grounded nor connected to external circuit), only electrons exist on the metal surface because the diffusion coefficient for electrons is much larger than that for ions, namely, the electrons move faster than ions. Although the metal surface covered by the sample was not exposed to plasma, there will be electrons distributed underneath since the electrons are free to move in metal, hence the potential at that spot will be the same as elsewhere of the metal. Therefore the Poisson equation can be written as

$$\begin{cases} \frac{d^2\varphi}{dz^2} = \frac{en_{2D}\delta(z)}{\varepsilon_r\varepsilon_0}, if\ z \le t_{AAO} \\ \frac{d^2\varphi}{dz^2} = \frac{e}{\varepsilon_0}\left[n_{e0} exp\left(\frac{e\varphi}{k_B T_e}\right) - n_{i0} exp\left(-\frac{e\varphi}{k_B T_i}\right)\right], if\ z > t_{AAO} \end{cases} \quad (1)$$

where n_{2D} is the electron density on the metal surface, t_{AAO} is the thickness of the AAO, and the metal surface is located at the position where $z = 0$.

Before the charged particles accumulate on the dielectric surface, the boundary conditions for the equations are $\varphi_{z=0} = \varphi_S < 0$, $\varphi_{z\approx t_{AAO}}|_{z<t_{AAO}} = \varphi_{z\approx t_{AAO}}|_{z>t_{AAO}}$ and $\varphi_{z\to\infty} = 0$. The solution for the equations above can be obtained as follows.

$$\begin{cases} \varphi = \varphi_S + \frac{en_{2D}z}{\varepsilon_r\varepsilon_0}, if\ z \le t_{AAO} \\ \varphi = \left(\varphi_S + \frac{en_{2D}t_{AAO}}{\varepsilon_r\varepsilon_0}\right) exp\left[-\frac{(z-t_{AAO})}{\lambda_D}\right], if\ z > t_{AAO} \end{cases} \quad (2)$$

One Debye length is considered as the sheath width in the following discussion for simplification, although the sheath width is conventionally defined as several Debye lengths.

For the case of metal only in plasma, it's convenient to consider the sheath region as a parallel plate capacitor with the plate-distance of λ_D and the total charge density of $-en_{2D}$. The capacitance is therefore $C = \varepsilon_0/\lambda_D = -en_{2D}/\varphi_s$. Substituting this relation into the first formula in equation (2) gives $\varphi = \varphi_s + \frac{en_{2D}z}{\varepsilon_r\varepsilon_0} \approx \varphi_s\left(1 - \frac{z}{\varepsilon_r\lambda_D}\right)$. Meanwhile, the potential attenuation on metal surface only in plasma can be simplified as $\varphi = \varphi_s exp\left(-\frac{z}{\lambda_D}\right) \approx \varphi_s\left(1 - \frac{z}{\lambda_D}\right)$ using Taylor expansion. Comparing these two relations, it can be found that the thin dielectric has the ability to *slow down* the potential attenuation in plasma by the factor of ε_r and therefore *extends* the weakened sheath on the top of the dielectric. The green dashed curve in Figure S3 describes the phenomenon, where $t_{AAO} = 5\lambda_D$.

3. The plasma sheath on the dielectric sitting on the metal surface after the charges accumulate on the dielectric surface

However, since the weakened electric field on dielectric surface can't provide enough ion flux to 'counteract' the electron flux, more electrons will diffuse to and be trapped on the dielectric surface. Therefore the ambipolar field will be re-established, and will have the same strength regardless the background potential coming from the metal stage, because it has to reach the intrinsic value of ambipolar field to keep the electron and ion fluxes balanced. An additional boundary condition should be introduced, which is $\frac{\partial\varphi}{\partial z}_{z\approx t_{AAO}}|_{z>t_{AAO}} = -E_A|_{z\approx t_{AAO}}$. The solution can be obtained as follows.

$$\begin{cases} \varphi = \varphi_s + \frac{(\varphi_0-\varphi_s)z}{t_{AAO}}, if\ z \le t_{AAO} \\ \varphi = \varphi_0 exp\left[-\frac{(z-t_{AAO})}{\lambda_D}\right], if\ z > t_{AAO} \end{cases} \quad (3)$$

The purple curve in Figure S3 indicates this re-establishment of plasma sheath, where $t_{AAO} = 5\lambda_D$.
